# Hunting for Helminths: short- and long-read shotgun metagenomics for helminth detection in faecal samples

**DOI:** 10.1371/journal.pntd.0014130

**Published:** 2026-09-21

**Authors:** Katie O’Brien, Ajay Elamaran, Mehmet Dayi, Georgia Keeling, William D. Nevin, Yuchen Liu, Mark Viney, Kieran Reynolds, Charmaine Bishop, Banchob Sripa, Menebere Woubshete, Pamela Sachs Nique, Rhiannon Wright, Jane Younger, Vicky L. Hunt

**Affiliations:** 1 Life Sciences Department, University of Bath, Bath, United Kingdom; 2 Forestry Vocational School, Düzce University, Konuralp Campus, Düzce, Türkiye; 3 Department of Clinical Sciences, Liverpool School of Tropical Medicine, Liverpool, United Kingdom; 4 Academic Department of Military Medicine, Royal Centre for Defence Medicine, Birmingham, United Kingdom; 5 Department of Evolution, Ecology and Behaviour, University of Liverpool, Liverpool, United Kingdom; 6 Tropical Disease Research Center, Department of Tropical Medicine, Faculty of Medicine, Khon Kaen University, Khon Kaen, Thailand; 7 Institute for Marine and Antarctic Studies, University of Tasmania, Hobart, Australia; Wellcome Sanger Institute, UNITED KINGDOM OF GREAT BRITAIN AND NORTHERN IRELAND

## Abstract

Soil-transmitted helminths (STHs) pose significant challenges to public health in endemic areas, necessitating reliable methods for their detection. Shotgun metagenomics enables simultaneous detection of STHs and microbes in a sample without prior knowledge of what is present. However, validation of shotgun metagenomics with known infection intensity or across different sequencing platforms has not been carried out for eukaryote parasites including STHs, and false positives remain a pervasive issue. We validated shotgun metagenomics as a method of STH detection in faecal samples. Using the *Strongyloides ratti* laboratory model of a STH infection we investigated how analytical methods (nucleotide-nucleotide matching, nucleotide-protein matching, marker gene detection, mitochondrial mapping), infection intensity (low and standard laboratory doses) and sequencing technology (short-read vs. long-read) affects sensitivity and specificity of detection. *S. ratti* was accurately detected at a standard laboratory dose, but low intensity infection were more difficult to detect. Only mitochondrial sequence mapping was 100% accurate at identifying *S. ratti* with no false positives. Overall, short-read outperformed long-read sequencing methods. We applied the same analytical methods to human faecal samples with confirmed infections for at least one of four STHs. Mitochondrial sequence mapping was also the most effective method for detecting STHs in human faecal samples, detecting 100% of *Necator americanus* and 92% of *Ascaris* spp. infections, but could not reliably detect STHs where DNA levels are expected to be low or variable. In conclusion, mitochondrial mapping was the most effective method of detection for sensitivity and specificity in both the laboratory system and human faecal samples. Our findings indicate that shotgun metagenomics should be approached cautiously using validated methods, particularly when infection intensity or DNA levels are expected to be low.

## Author summary

Soil-transmitted helminths (STHs), such as the parasite *Strongyloides*, are important gastrointestinal parasites of humans and livestock. Accurate methods of detection for diagnostics and monitoring are important to implement suitable control and treatment strategies. Here we validate a shotgun metagenomics approach, where all DNA in a sample is sequenced, for detecting STHs in faecal samples using a *Strongyloides* laboratory model for infection. *Strongyloides* was reliability detected in faecal samples at higher infection levels, but mitochondrial genome mapping of the sequences was the only analytical method that reliably detected *Strongyloides* at lower infections levels. These results were reflected in faecal samples from humans infected with STHs, where mitochondrial mapping was also the most reliable method. However, species that were associated with low levels of parasite material or DNA in the faeces including *Strongyloides stercoralis,* were more difficult to detect. We compared two sequencing methods: short-read (SR) Illumina and long-read (LR) Oxford Nanopore Technologies, but SR outperformed LR shotgun metagenomics. Contamination of bacterial sequences in parasite genome assemblies was problematic for analysis and contributed to false positive results. Future work should focus on specific targeting of eukaryote DNA either at the laboratory or bioinformatic stage to improve STH detection further.

## Introduction

Soil-transmitted helminths (STHs) infect 1.5 billion people globally, particularly in Low- and Middle-Income Countries. STHs are recognised by the WHO as causative agents of Neglected Tropical Diseases (NTD) [1]. Despite the progress that has been made in reducing both transmission and morbidity caused by STHs through preventative chemotherapy, detection of STH infection remains a challenge.

Coproscopic methods of STH detection [2] are considered generally reliable [3,4] and low cost [5], but they require specialised technicians, are time consuming and low throughput. At low infection burdens, as is often observed in natural infections, low sensitivity associated with coproscopy can be problematic [6–9]. This can result in underestimated prevalence, particularly for some key species such as the STH *Strongyloides stercoralis* [10]. Molecular techniques such as loop-mediated isothermal amplification (LAMP) assays and PCR/RT-PCR are more sensitive and have begun supplementing conventional STH detection methods [11–15]. However, they are costly, can require *a priori* knowledge of the target species [16], face challenges such as taxon-specific PCR amplification biases [17,18] and a lack of resolution for species-level identification [19–21]. STHs commonly occur as co-infections [22] and symptoms cannot be used to determine the agent of disease [23,24]. An untargeted method of identification is particularly important where reliable species-level identification is required for tracking parasite transmission, disease surveillance, detecting newly introduced species or variants, monitoring control programmes and outbreaks, and evaluation of treatment efficacy [25].

Shotgun metagenomics sequences all DNA in a sample, allowing for simultaneous characterisation of eukaryotic, bacterial and viral diversity, without prior knowledge of the organisms which are likely to be present. As such, shotgun methods have the potential to detect multi-STH infections and allow for exploration of the microbiome, including bacterial infections. The use of shotgun metagenomics for detecting microbes is well-established [26–30], benchmarked, and validated [31–37]. False positive reporting is a serious issue in bacterial pathogen detection, and guidelines to ensure accuracy have been published, highlighting the importance of validating methods [38,39]. Shotgun metagenomics for eukaryote parasite detection lacks validation, and analytical methods are not well established [40–46]. Studies have mostly relied on confirmation of parasite infection by other methods of detection, such as microscopy or targeted PCR, each of which require independent validation for sensitivity and specificity. Infection intensity is also either unknown or estimated for most validation methods limiting our understanding of whether low-intensity infections are being reliably detected [19,47–54]. Among the major challenges in using shotgun metagenomics for STH detection are: (i) STHs are present at lower abundance (i.e., smaller fraction of reads in sequencing libraries) than microbes in faecal samples, making them more difficult to detect [55]; (ii) eukaryotic genome assemblies are commonly contaminated with bacterial sequences, which can lead to false positive identifications [56–58]. Work by Papaiakovou *et al* (2023) [48] demonstrated that shallow shotgun sequencing paired with mitochondrial genome mapping is an effective approach for identifying STHs in faecal samples. This approach leverages the high copy number of mitochondrial genomes to improve sensitivity, even when parasite DNA is present at low abundance within total faecal DNA. The availability of complete mitochondrial genomes for many STHs also enables more accurate taxonomic assignment and can support phylogenetic and population genetic analyses. However, performance can be influenced by variation in sample processing and DNA extraction methods and relies on the completeness of reference mitochondrial genome databases.

The potential of long-read (LR) shotgun metagenomic sequencing with Oxford Nanopore Technologies (ONT) or Pacific Biosciences to simultaneously detect and identify eukaryotes and microbes remains largely unexplored^,^ [59]. These methods offer advantages of portability and real-time data generation, and could aid detection of STHs that lack high quality genome assemblies or where differentiation of genotypes is important, by identifying longer sequences [60]. The cost of both LR and short-read (SR) metagenomics is reducing, presenting feasible diagnostic tools for the future [61,62], as well as use as a monitoring and research tool.

In this study we compare the mitochondrial mapping method [48] with three further analytical methods of shotgun metagenomics using SR (nucleotide-nucleotide matching, nucleotide-protein matching, marker gene detection, mitochondrial mapping), and further compare LR, and hybrid LR-SR sequencing technologies, which have not previously been validated for STH detection. We use a laboratory system of rats infected with known infection intensity of *Strongyloides ratti,* an established laboratory model for *Strongyloides* infection [63]. STH detection using shotgun metagenomics is assessed on the analytical approach used, infection intensity (standard and low dose) and sequencing technology (SR and LR). We further apply these methods to human faecal samples infected with one of four STHs and assess the sensitivity and specificity of the methods tested.

## Methods

### Ethics statement

Rodent work was performed under the authority of licences issued by the Animals (Scientific Procedures) Act 1986 and was approved by the University of Bath Animal Welfare and Ethical Review Body. Collection of samples from human participants was carried out after either written or oral consent. The study was approved by the Khon Kaen University Ethics Committee for Human Research (reference HE641605) and the Central University Research Ethics Committee at the University of Liverpool (reference 10936).

### Maintenance of *Strongyloides ratti*

Five week old female Wistar rats were infected with *S. ratti* ED321 by subcutaneous injection of third stage infective larvae (iL3) prepared from faecal cultures as described by Hunt et *al*. [64] Rats were infected with either 50 (low dose) or 500 (standard dose) iL3s suspended in 200 μL of PBS. Controls were injected with PBS only.

#### Sample collection and preparation.

***S. ratti***: Rat faeces were collected from rats between 6–11 days post infection (dpi) and stored at -80°C until extraction. DNA extraction was carried out using the QIAamp PowerFecal Pro DNA kit (Qiagen) according to the manufacturer’s instructions (S1 Methods).

***S. stercoralis***: Four human faecal samples (CKH130, CKH21, LWH76, KKU01) were collected from participants in Thailand (Table 1) as described by Viney et *al*. [65]

**Table 1:** *Strongyloides stercoralis*-positive samples collected from Thailand.

Sample ID	Sample Site	Age	Gender	No. iL3s
CKH21	16°07’53“N 102°39’01”E	71	Male	>1000
LWH76	16°07’53“N 102°39’01”E	62	Male	>500
CKH130	16°07’53“N 102°39’01”E	50	Male	45
KKU01	16°28’11“N 102°49’52”E	Unknown	Unknown	53

Three further samples were collected from patients in the UK who had recently migrated from Fiji (infective larvae numbers present in faeces are unknown for these samples). All human participants were confirmed to have a *S. stercoralis* infection using microscopy or PCR (S1 Methods).

### Illumina SR sequencing

DNA fragment libraries from standard dose rat faeces were constructed with NEBNext Ultra II FS DNA kit (1/2 volume protocol). DNA fragment libraries from low dose infection rat faeces and human faecal samples (Thailand and Fiji) were constructed using the Eurofins Genomics extraction kit. Sequencing was carried out on a NovaSeq SP (2x150bp), generating ~10-12.9 million (rat) and ~41.5 million (human) paired end reads per library (S1-S4 Tables). Host genome sequences for humans (*Homo sapiens* genome assembly GRCh38_noalt_as, NIH accession GCF_000001405.26) and rats (*Rattus norvegicus* Rnor 6.0, NIH accession GCF_000001895.5) were removed by mapping raw reads to the host genome using Bowtie2 (v. 2.4.2) [66] and Samtools (v. 1.1.2) [67]. The unmapped reads were extracted from the resulting BAM file using Bedtools (v. 2.29.2) [68] ‘bamtofastq’ and converted from BAM files into fastq.

### ONT sequencing and pre-processing

A subset of three samples (one infected 11dpi standard dose, one infected 11dpi low dose and one control 11dpi) used for SR sequencing were selected for LR metagenomic sequencing using MinION sequencing technology from Oxford Nanopore Technologies (ONT) (S1 Fig). Samples were prepared for sequencing using the SQK-LSK110 Ligation Sequencing Kit according to the manufacturer’s instructions in combination with the following NEBNext products: NEBNext FFPE Repair Mix (M6630), NEBNext Ultra II End repair/dA-tailing Module (E7546) and the NEBNext Quick Ligation Module (E6056). Samples were sequenced for up to 72 hours on a FLO-MIN106 flow cell connected to a laptop hosting MinKNOW software. The fast5 files generated from sequencing were converted into fastq format using the high accuracy version of Oxford Nanopore’s GPU-enabled ‘Guppy’ program with the following parameters: flowcell:‘FLO-MIN106’, kit:‘SQK-LSK110’, callers:14, gpu runners per device:8, chunks per runner:768, chunk size:500. Quality control assessment of the MinION run was performed using MinIONQC [69] and FASTQC [70], and the first 35 bases of each sequencing read were removed due to low quality using NanoFilt [71]. All reads greater than 54,000 bases in length were also removed due to low quality. Following trimming, reads were processed by Nanoplot [71], and sequences => Q7 were retained for further analysis. Host genome sequences (from same source as SR analysis) were removed using Minimap2 with the ‘ax map-ont’ preset [72] and the resulting unmapped reads were converted into fastq files as previously mentioned (S5 Table).

### Detection of *S. ratti* using SR metagenomics

Taxonomic assignment was compared using four analytical tools: Kraken2 (nucleotide-nucleotide matching) [73], DIAMOND+MEGAN (nucleotide-protein matching) [74], EukDetect (marker genes) [49], and mitochondrial mapping [48] (S1 File).

- K-mer based nucleotide-nucleotide matching with Kraken2

A custom Kraken2 nucleotide reference database was constructed using the ‘kraken2-build’ command. The standard RefSeq libraries supplied by Kraken2 for fungi, archaea, bacteria, viruses, and protozoa were first downloaded using the ‘kraken2-build --download-library’ command. Following this, the reference sequences for the host (*Rattus norvegicus* - Rnor6.0) and its main dietary components (wheat: IWGSC CS RefSeq v2.1, barley: MorexV3_pseudomolecules_assembly, soy: Glycine_max_v4.0 and maize: Zm-B73-REFERENCE-NAM-5.0) were downloaded from NCBI and added to the database using the ‘kraken2-build --add-to-library’ command. The genomes for *S. ratti* and five closely related species, alongside a further eight nematode genomes that span multiple evolutionary clades of Nematoda, were also added to the database. The nematode genomes included were: *S. ratti, S. stercoralis, Parastrongyloides trichosuri, S. papillosus, S. venezuelensis, Rhabditophanes sp. KR3021, Meloidogyne hapla, Bursaphelenchus xylophilus, Ascaris suum, Brugia malayi, Trichuris muris, Trichinella spiralis, Necator americanus* and *Caenorhabditis elegans* (S6 Table). The addition of these genomes enabled the assessment of false positive hits to non-target genomes. All nematode genomes were downloaded from the WormBase parasite database V16.0 [75] (accessed 13/05/2022). The final custom database was constructed using the ‘kraken2-build --build’ command.

Kraken2 classification was applied to all short reads against the custom database, using the ‘--report’, ‘--classified-out’, and ‘--gzip-compressed’ options. All Kraken2 ‘report’ output files were combined into a biom file alongside the relevant metadata using kraken-biom (v. 1.0.1). The biom file was imported into R (v. 4.2.1) using the ‘import-biom’ function from phyloseq v.1.46.0 [76].

A custom R script was run to prevent erroneous taxonomic assignments due to taxonomic revisions (https://github.com/Vicky-Hunt-Lab/Metagenomics_tax_update). This involved taking the taxa IDs from the OTU table and reclassifying them according to current NCBI taxonomy using the ‘classification’ function from the rentrez package (v.4.3.2) [77] in R, e.g., all entries classified as phylum “Firmicutes” were reclassified to “Bacillota”. An abundance filter was applied to remove any observations reaching less than 0.001% of total abundance.

Taxonomic composition was visualised using ggplot2 (v.3.3.6) [78] in R. To investigate the rate of true positive assignment of nematode taxa using Kraken2, any hits to the phylum “Nematoda” were filtered, and reads not aligning to a specific species were discarded.

ii. Nucleotide-protein matching with DIAMOND+MEGAN pipeline

We followed the methods outlined in Bağcı et *al*. [74] where SR sequencing outputs were translated, then aligned against the NCBI-nr database using the ‘blastx’ function in DIAMOND (v. 2.0.15) [79], followed by taxonomic binning with MEGAN community edition (v. 6.24.11) [74]. DIAMOND’s double-indexing approach is approximately 20,000 times faster than a regular blastx search [79]. The nr database was downloaded from NCBI (12^th^ May 2022), and a DIAMOND index was created using the ‘diamond makedb’ command. After the concatenation of paired-end samples, the ‘diamond blastx’ command was used to produce a diamond archive file (.daa). The MEGAN database (megan-map-Feb2022.db) and tools suite were downloaded from the MEGAN website [80]. The ‘daa-meganizer’ function categorised reads into taxonomic bins. Alignments were converted for analysis using the pb-metagenomics-tools script ‘Convert_MEGAN-NCBI-c2c_to_kreport-mpa.py’ (https://github.com/PacificBiosciences/pb-metagenomics-tools). The “megan-c2c.kreport.txt” output files were combined into a biom file alongside the relevant metadata using kraken-biom (v. 1.0.1) and was imported into R as described previously.

Eukaryotic reads were extracted from the.daa files using the ‘read-extractor’ function from MEGAN, followed by rerunning the pipeline with the ‘--iterate’ option for increased sensitivity in the blastx search. The minimum support percentage for taxonomic assignment after eukaryotic extraction was adjusted from the default of 0.01% to 0.1% to provide more conservative assignments. All outputs from the eukaryotic extraction step were again combined into a biom file, and any species-level alignments to taxa falling under the phylum “Nematoda” were retained and imported into R as described previously. The DIAMOND+MEGAN pipeline was not feasible for high depth sequencing analysis due to computational demands (S1 Methods). As such, we do not recommend using this method for high depth sequencing where samples generate more than 50 million reads.

iii. Eukaryote marker gene detection with EukDetect

To investigate whether species-specific markers could be used in the identification of *S. ratti*, EukDetect (v. 2, 14^th^ October 2022) was used [49]. Of the > 500,000 markers included in the database, approximately 17,000 are derived from >150 species of nematode or platyhelminth, including 13 of the 14 nematodes chosen for inclusion in the custom Kraken2 database (*A. suum* is not included). The EukDetect database was downloaded from Figshare (v. 9) [81] and the analysis was conducted using the ‘runall’ mode on all short reads.

iv. Mitochondrial mapping

In our Kraken reference database, only three nematode species - *S. stercoralis*, *T. muris*, and *T. trichiura* - had mitochondrial genomes included with their nuclear reference assemblies when downloaded from their repository source. To evaluate whether mitochondrial mapping is a valid detection approach, we therefore performed a separate analysis. Mitochondrial mapping was performed as per Papaiakovou et *al*. [48], with the exception that Bowtie2 [66], rather than BWA-MEM [82], was used for mapping reads, and mitochondrial genomes for an additional eight species (*S. papillosus, S. ratti, S. venezuelensis, P. trichosuri, Rhab sp. KR3021, B., C. elegans, T.* muris) were extracted from WormBase ParaSite v16 and included. Analysis in R followed the script https://github.com/MarinaSci/IJP_genome_skimming. Alignments to genomes are reported as “normalised coverage” which refers to the number of reads mapped per million reads generated per mitochondrial genome size (Mb).

A normalised “reads aligned per million reads generated” value was created for all species-level nematode alignments (RPM), to adjust for variation in read counts across samples for all Kraken2, DIAMOND+MEGAN and EukDetect outputs, alongside the “Normalised coverage” values generated using mitochondrial mapping (S1 File). Heatmap visualisation for all four bioinformatic methods were generated using ggplot2 in R. Wilcoxon rank sum tests were conducted to compare the RPM of alignments between various sample groups.

Note: A comparison of computational effort between all four methods applied here can be found in S1 Methods.

### Taxonomic assignment and detection of *S. ratti* using LR shotgun metagenomic reads

We used the following tools for LR data analysis: i) nucleotide-protein matching with DIAMOND+MEGAN-LR [74], ii) Assembly-based alignment with the CZ ID online platform [83], and iii) mitochondrial mapping.

- Nucleotide-protein matching with DIAMOND+MEGAN pipeline

The DIAMOND+MEGAN pipeline was used for LR analysis with alterations to account for longer reads. Firstly, we applied a recommended penalty for frameshifts in DNA-vs-protein alignments (‘-F 15’). We also added the parameters ‘--range-culling’ and ‘--top 10’, enabling the filtering of alignments below a certain bit score, thus retaining only the most relevant hits across a query sequence. Diamond archive output files were meganized using ‘daa-meganizer’ with the ‘--longReads’ parameter so that the number of aligned bases, rather than reads, would be used to assign taxonomy. As a result, all taxonomic assignments for LR data are reported as “number of bases per million reads” (BPM). An abundance filter was applied to remove any observations reaching less than 0.001% of total abundance. Eukaryote read extraction, and conversion into biom files remains the same as for short reads.

ii. Assembly-based alignment with CZ ID online platform

LR sequences were uploaded to CZ ID [84], where they were profiled using the mNGS nanopore pipeline. Species-level taxonomic assignments were imported into R and taxonomic categories were reformatted as described previously (https://github.com/Vicky-Hunt-Lab/Metagenomics_tax_update) and BPM values were generated (S2 File).

iii. Mitochondrial mapping

Mitochondrial mapping was performed as per Papaiakovou et *al* [48]. For LR samples, both Bowtie2 and Minimap2 [72] were used to map reads, to assess performance using a SR aligner (Bowtie2) compared to a LR aligner (Minimap2). Analysis in R followed the script in https://github.com/MarinaSci/IJP_genome_skimming. Alignments to genomes are reported as “normalised coverage” which refers to the number of reads mapped per million reads generated per mitochondrial genome size (Mb) (S2 File).

#### Detection of *S. ratti* using hybrid metagenomic assemblies.

For the three samples that had both SR and LR sequencing data (Standard Dose 11dpi, Low dose 11dpi, Control 11dpi), hybrid assemblies were generated using metaSPades v3.15.5 with default options [85]. Assemblies were further evaluated by metaquast v5.2.0 with default options [86] and contigs were grouped into bins using MetaBAT2 v2.12.1. with default options [87] where each bin corresponds to an individual genome. Assembly bins were evaluated for contamination, completeness and N50 values with CheckM v1.2.2 [88], and those with contamination <10% and completeness >90% were considered complete genomes (S7 Table). Three files were generated from hybrid assembly (i) a file containing all assembled contigs including unassembled reads, (ii) a MetaBAT2 binned file containing bins clustered into putative genomes, (iii) a MetaBAT2 unbinned sequence file containing all sequences not assigned to a genome bin. The assembled contigs for each sample were aligned against a custom Kraken2 database as mentioned previously. Nematode classifications were normalised to RPM S2 File). Mitochondrial mapping was also performed as mentioned previously for LRs, using both Bowtie2 and Minimap2, to generate normalised coverage values (S2 File).

A custom BLAST database was created using the “makeblastdb” command with default parameters comprising the Rnor 6.0 genome, the *S. ratti* genome, and 13 other nematode species from four evolutionary clades including free-living and parasitic nematodes, as included in the Kraken2 custom database. As all binned sequences had microbial origins (S3 File), the BLAST search was performed on unbinned sequences with the ‘blastn’ command, using an e-value threshold of 0.001 and a percent identity of 95% (S4 File). In the first instance, the BLAST search was carried out only on sequences greater than 1kb in length. Subsequently, another BLAST search was also performed on sequences less than 1kb in length to do a comparative analysis.

#### Sensitivity, specificity rates.

Measures of sensitivity, specificity, precision, false positive detection rate (FPR), and F1 scores for SR (S5 File), LR and LR-SR hybrid analysis (S6 File) were calculated as below:

Sensitivity = True Positives/(True Positives + False Negatives)

Specificity = True Negatives/(False Positives + True Negatives)

Precision = True Positives/(True Positives + False Positives)

FPR = 1 – Specificity

F1 Score = 2 * (Sensitivity* Precision)/ (Sensitivity + Precision).

### Parasite detection using SR metagenomics on human samples

We reanalysed 24 metagenomic shotgun samples (SRA, BioProject PRJNA407815) from a previously published microbiota-parasite interaction study [89]. Kraken2, EukDetect, and mitochondrial mapping were applied as described above for SR analysis of *S. ratti* in rat faecal samples (S7 File). DIAMOND+MEGAN was not used because it was the least accurate for *S. ratti* samples (S5 File) and computationally more demanding (S1 Methods). Results were compared with qPCR or formal-ether results (S8 Table) for *N. americanus*, *Ascaris spp*., and *T. trichiura* infections. True-positive RPM detection rates, using sensitivity, specificity, precision, false positive detection rate, and F1 scores, were calculated as described above (S8 File).

The following adjustments were made prior to analysis of human faecal samples: 1) trimmomatic v. 0.39 [90] was used to remove any unpaired or short (<20 bp) reads, 2) An alignment length filter of 90 bp, rather than the default 130 bp, was used for mitochondrial mapping, as the files uploaded to SRA had already undergone trimming, 3) The genomes for *A. lumbricoides* and *T. trichiura* were added to the custom Kraken2 database, 4) Any reference genome sequences with length <10 kb were filtered from the Kraken2 reference database. We did this as per Papaiakovou et *al.*[48], where removal of short scaffolds (<10kb) was shown to effectively remove bacterial contamination from the reference genome of *Enterobius vermicularis* and 5) A minimum threshold of 50 RPM was applied to Kraken2 outputs based on our SR analysis in controlled lab settings. The *A. lumbricoides* genome was downloaded from WormBase Parasite V16.0 and the *T. trichiura* genome was sourced from Doyle et *al.*[91].

We extended our analyses to include the detection of *S. stercoralis* in seven confirmed positive samples collected from humans in Thailand (n = 4) and from participants of a clinical cross-sectional study on Fijian migrants to the United Kingdom (n = 3). Kraken2, EukDetect, and mitochondrial mapping methods were used to assign the presence of *S. stercoralis*, using the same custom Kraken2 reference database as in the previous section (with a threshold of 50 RPM and removal of <10kb scaffolds).

## Results

We assessed the importance of the following parameters for detection of *S. ratti* DNA in rat host faecal samples using shotgun metagenomics.

(i) Computational analysis: four approaches of taxonomic assignment were used to identify *S. ratti* DNA: 1) Kraken2, a k-mer based nucleotide-nucleotide matching tool [73], 2) MEGAN+DIAMOND, a nucleotide-protein matching tool [74], 3) EukDetect, a eukaryote marker gene detection tool [49], and 4) mitochondrial genome mapping [48].
(ii) Infection intensity: a standard laboratory dose (500 iL3) compared with a low dose (50 iL3) infection.
(iii) Sequencing technology: SR shotgun metagenomics compared with ONT LR shotgun metagenomics and hybrid assembly.

### *S. ratti* is detected at standard infection dose using SR shotgun metagenomics

The majority of reads were classified as ‘bacteria’ and only 0.35% and 0.064% of reads were identified as ‘nematode’ using Kraken2 [73] (Fig 1a) and DIAMOND+MEGAN [74], respectively (Fig 1b). Kraken2 [73], DIAMOND+MEGAN [74], EukDetect [49] and mitochondrial genome mapping [48] all accurately identified *S. ratti* in the SR shotgun metagenomic sequencing data from faeces of rats infected with a standard laboratory dose. False positive assignment of reads occurred at low levels using Kraken2 and DIAMOND+MEGAN, but this was resolved by implementing a threshold to remove sequences with <50 reads per million (RPM). All methods had high specificity and sensitivity for detecting *S. ratti* and did not detect false positives (Fig 1c-1f, S1 and S5 Files).

**Fig 1:**
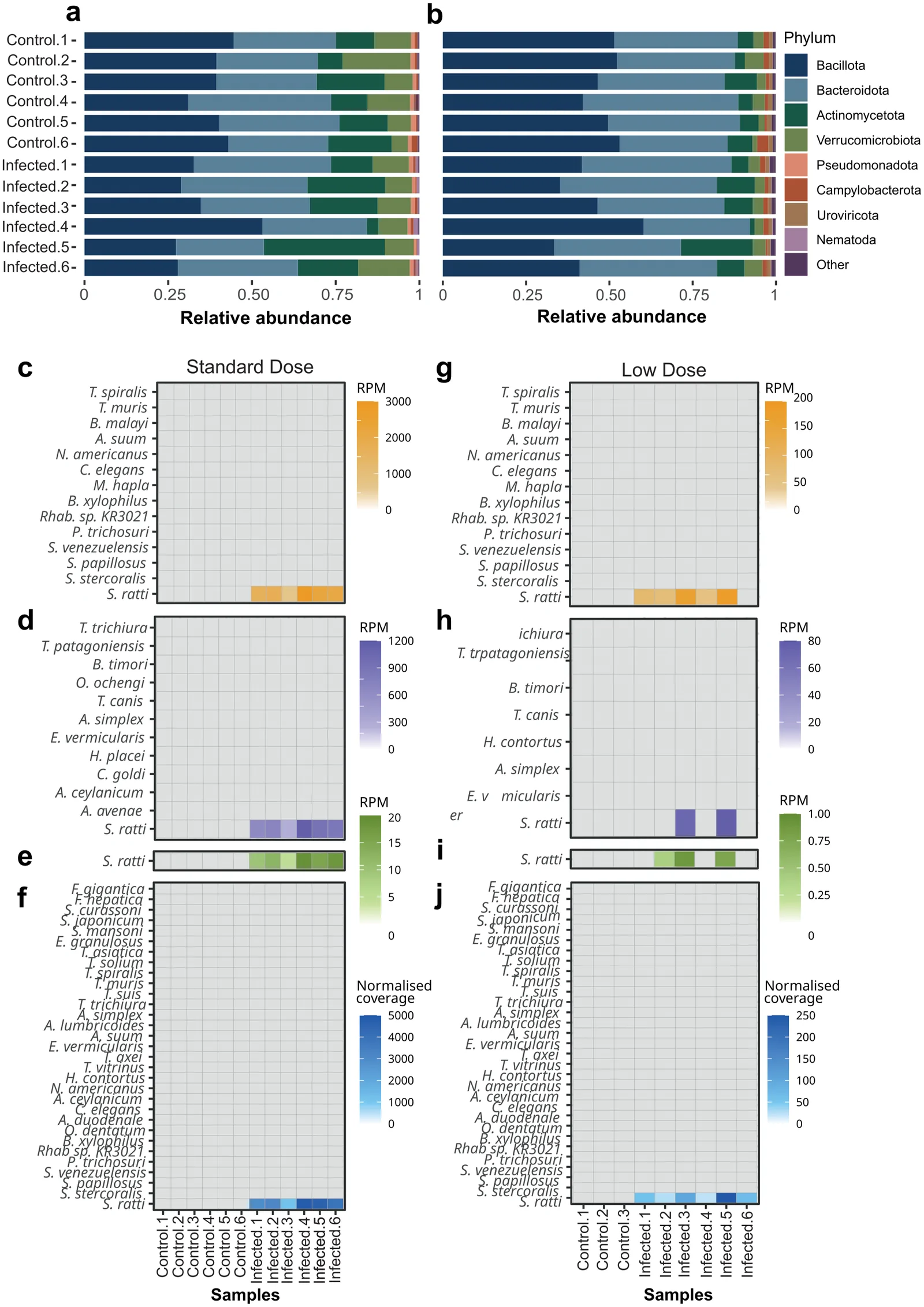
Taxonomic classification and *S. ratti* detection in rat faecal samples using SR shotgun metagenomics. **(a-b)** Relative abundances of core phyla (>0.001% abundance) in rat faecal samples infected with a standard dose of *Strongyloides ratti* (500 iL3s) with an average sequencing coverage of ~10.5M reads, identified using (**a**) a custom Kraken2 database and (**b**) the DIAMOND+MEGAN pipeline. Low abundance phyla are grouped into the “Other” category. (**c-f**) assignment of reads to nematodes from SR shotgun metagenomics rat faecal samples from a standard dose infection of *S. ratti* using (**c**) a custom Kraken2 database containing 14 nematode genomes with a 50 RPM threshold for detection, (**d**) the DIAMOND+MEGAN pipeline with a 50 RPM threshold for detection, **(e)** EukDetect marker gene detection and (**f**) mapping against 31 nematode mitochondrial genomes. **(g-j)** Assignment of reads to nematodes from SR shotgun metagenomics rat faecal samples from a low dose infection of *S. ratti* using (**g**) a custom Kraken2 database containing 14 nematode genomes with a 50 RPM threshold for detection, (**h**) the DIAMOND+MEGAN pipeline with a 50 RPM threshold for detection, **(i)** EukDetect marker gene detection and (**j**) mapping against 31 nematode mitochondrial genomes. Species are ordered by increasing relatedness to *S. ratti*. Colour on the heatmap denotes level of detection, with grey squares indicating no detection. RPM = Reads mapped per million reads; Normalised coverage = Reads mapped per million reads per genome size (Mb).

### *S. ratti* can be detected at low infection intensity using mitochondrial mapping

To test if infection intensity is important for *Strongyloides* detection we compared SR shotgun metagenomics data from rat faecal samples for a standard dose (500 iL3) to a low dose (50 iL3) of *S. ratti* infection. *S. ratti* was detected at lower levels in samples with lower infection intensity (Wilcoxon rank sum test, W = 36, p < 0.01) (S9 Table).

Kraken2, DIAMOND+MEGAN and EukDetect produced false negatives, detecting *S. ratti* in 5/6 (Fig 1g, F1 = 0.91, FPR = 0%), 2/6 (Fig 1h, F1 = 0.5, FPR = 0%) and 3/6 (Fig 1i, F1 = 0.67, FPR = 0%) low dose infection samples, respectively (S1 and S5 Files). As with standard dose samples, a 50 RPM filter was implemented for Kraken2 and DIAMOND+MEGAN to reduce false positive assignment of reads. Reducing the filter threshold to 25 RPM for DIAMOND+MEGAN increased true positive detection of *S. ratti* (F1 = 0.67), but also increased false positive detection of non-target species (S5 File). *S. ratti* was correctly detected in all low dose infected samples using mitochondrial mapping and there was no false positive detection of non-target species or detection of *S. ratti* in the control samples (Fig 1j, F1 = 1, FPR = 0%, S5 File). Overall, low doses of infection lead to poorer *Strongyloides* detection, except for mitochondrial mapping methods which maintained sensitivity and specificity.

### SR metagenomics outperforms LR metagenomics and hybrid assembly for detection of *S. ratti*

We assessed the detection of *S. ratti* using ONT LR metagenomics with two approaches: (i) LR only (Fig 2), and (ii) LR-SR hybrid assembly (Fig 3). The same DNA samples were directly compared for LR and SR sequencing, so differences are due to the sequencing or analytical methods, not variation between samples.

**Fig 2:**
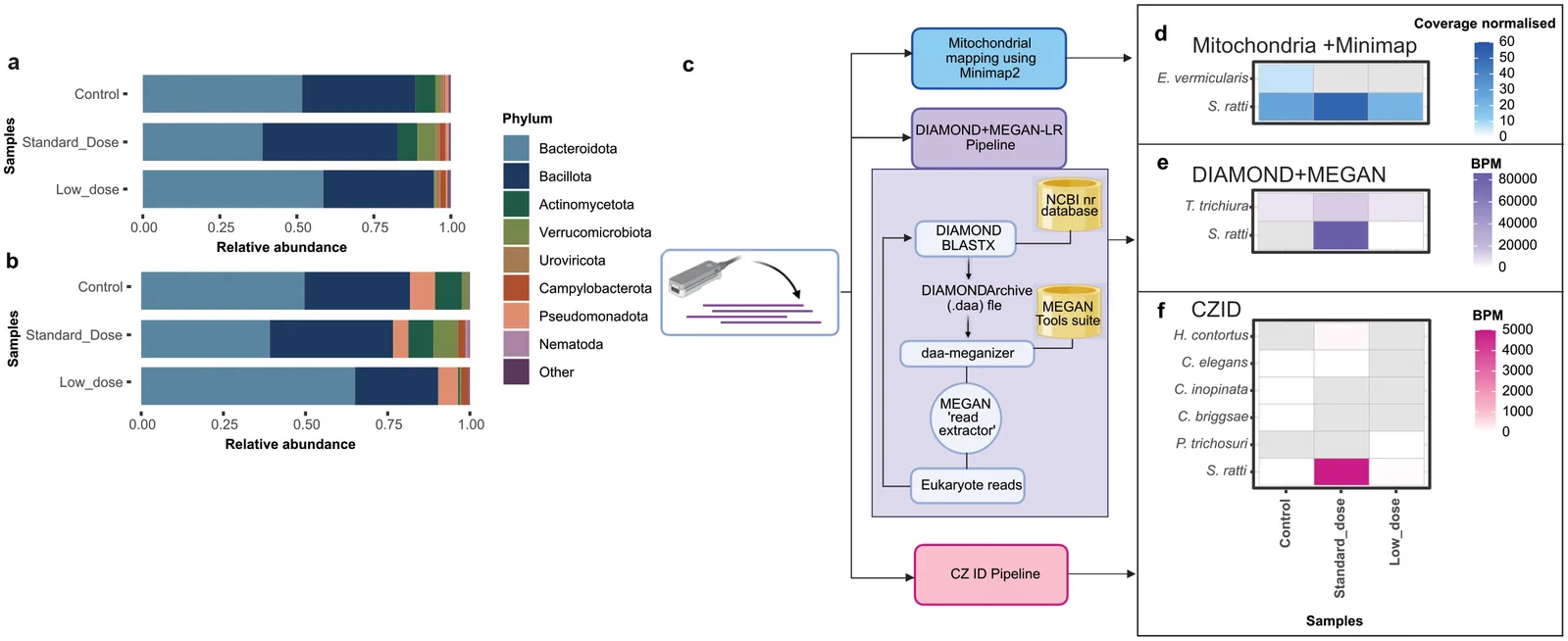
*S. ratti* Detection in Rat Faecal Samples Using LR Shotgun Metagenomics. (**a-b**) Relative abundances of core phyla (>0.001% abundance) in rat faecal samples infected with a standard (500 iL3s) or low dose (50 iL3s) of *S. ratti* using ONT LR sequencing, identified using (**a**) the DIAMOND+MEGAN-LR pipeline and (**b**) the CZ ID pipeline. Low abundance phyla are grouped into the “Other” category. (**c**) Overview of the bioinformatics pipeline used for nematode detection in LR shotgun metagenomics samples. (**d-f**) Nematode detection in LR shotgun metagenomics samples using (**d**) mitochondrial mapping with Minimap2, (**e**) the DIAMOND+MEGAN-LR pipeline and (**f**) the CZ ID pipeline. BPM = Number of bases per million reads; Normalised coverage = Reads mapped per million reads per genome size (Mb). Colour denotes level of detection, with grey squares indicating no detection. Created in BioRender. Hunt, V. (2026) https://BioRender.com/dt3xx5x.

**Fig 3:**
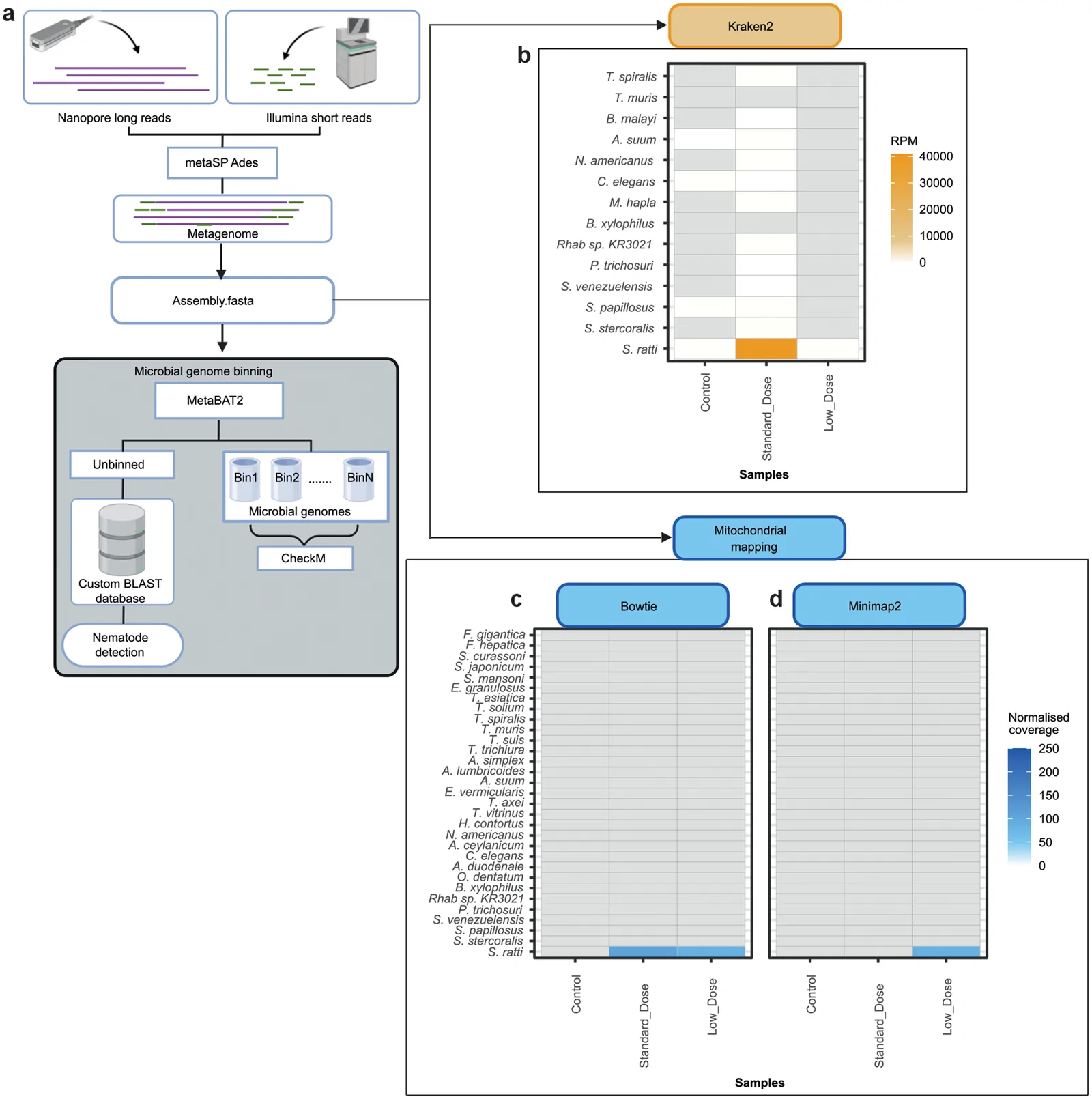
*S. ratti* Detection in Rat Faecal Samples Using LR-SR Hybrid Metagenomic Assembly. (**a**) Overview of the bioinformatics pipeline used for nematode detection using hybrid assemblies of LR-SR shotgun metagenomics. Four methods of detection were employed, using a custom BLAST database against unbinned assembled contigs, Kraken2, and mitochondrial mapping using Bowtie2 and Minimap2. (**c-d**) Nematode detection in hybrid metagenomic assemblies infected with a standard (500 iL3s) or low dose (50 iL3s) of *Strongyloides ratti*, using (**b**) a custom Kraken2 database containing 14 nematode genomes, (**c**) mapping against 31 nematode mitochondrial genomes with Bowtie2 and (**d**) Minimap2. RPM = Reads per million reads; Normalised coverage = Reads mapped per million reads per genome size (Mb). Colour denotes level of detection, with grey squares indicating no detection. Created in BioRender. Hunt, V. (2026) https://BioRender.com/0kb5o8u.

The LR data identified 0.1% and 0.3% ‘nematode’ reads for DIAMOND + MEGAN and CZ ID, respectively for a standard dose infection (Fig 2a and 2b). DIAMOND+MEGAN and CZ ID detected *S. ratti* at the standard and low dose (Fig 2e and 2f). However, false positive assignments to *T. trichiura* were observed in all LR samples using DIAMOND+MEGAN (Fig 2e). Five species were falsely identified by CZ ID and *S. ratti* was detected in control samples (Fig 2f). Applying a BPM threshold to eliminate false positives also lead to false negatives. Mitochondrial mapping with Bowtie2 produced no alignments to *S. ratti* in standard or low dose infections. Mitochondrial mapping with Minimap2 was also unsuccessful (F1 = 0), because the control sample falsely detected *S. ratti* in control samples and *E. vermicularis* (Fig 2d) (S2 and S6 Files).

A LR-SR hybrid assembly generated at least 445,183 contigs per sample and a total assembly size of >435 Mb/sample (S7 Table). As no standard method for assignment of assembled contigs has been established for eukaryotic sequences, we compared several approaches (Fig 3a):

(i) Kraken2 kmer approach: *S. ratti* was identified at 40,505 RPM for the standard infection dose (Fig 3b), and only 61 RPM for the low infection dose, a similar level to the false positive identification of *S. ratti* in the control sample (74 RPM), suggesting that this method is not suitable for detecting low intensity *S. ratti* infection.
(ii) Mitochondrial mapping: *S. ratti* was correctly identified using Bowtie2 (normalised coverage value standard = 109, low = 86.1). *S. ratti* was only identified in the low dose sample using Minimap2 (normalised coverage value = 86.1). Neither Minimap2 nor Bowtie2 mapping methods produced false positive alignments. Hybrid mitochondrial mapping outperformed LR only mitochondrial mapping (Hybrid: F1 = 1, Long: F1 = 0) (Fig 3c and 3d).
(iii) Genome assignment of contigs: Contigs were assigned to genomes (METABAT2, CheckM) and unassigned contigs were identified using BLAST: Assembled contigs were assigned to 291 (standard dose), 165 (low dose) and 143 (control) genomes, including 47 (standard dose), 15 (low dose) and 15 (control) full genome assemblies, and were all taxonomically assigned as microbes (S3 File) (S7 Table). The standard dose sample had only 46 hits to *S. ratti* for contigs >1kb in length, with alignments between 0.9 to 5.7kb (S4 File). No hits were identified for *S. ratti* in the low dose and control sample. Only hits for *S. ratti* and the host organism *Rattus norvegicus* were identified across samples (FPR = 0). Expanding our criteria to include sequences <1kb identified 15,144 and 30 *S. ratti* hits for standard and low dose samples, respectively. No *S. ratti* hits were identified in the control sample, but false positive detection of non-target nematodes were detected at low levels in all samples (S4 File). This method lacked sensitivity at low doses. Overall, LR ONT sequencing offered no advantage for detecting *S. ratti* compared with SR shotgun metagenomics.

### SR Shotgun metagenomics identifies *N. americanus* and *Ascaris spp.* in human faecal samples

We analysed previously published sequence data for 24 shotgun metagenomic samples of human faeces collected from Indonesia and Liberia [89] using EukDetect, Kraken2 (50RPM threshold) and mitochondrial mapping. Of the 24 samples, 15, 12 and 2 were qPCR-positive or formol ether concentration positive for *N. americanus, A. lumbricoides* and *T. trichiura*, respectively [89] (S8 Table).

Of the 15 confirmed *N. americanus* samples, Kraken2, EukDetect, and mitochondrial mapping methods correctly identified *N. americanus* in 11, 6 and 15 samples, respectively (Fig 4a). False positive assignment to *N. americanus* using Kraken2 occurred in 3 out of 24 samples (Fig 4a and 4b) (F1 score = 0.76, FPR = 33%). Mitochondrial mapping and EukDetect only detected *N. americanus* in qPCR confirmed samples (FPR = 0%), and produced F1 scores of 1, and 0.57 respectively. For low-intensity infections (based on qPCR), EukDetect performed the worst, failing to detect all three low-level samples; Kraken2 missed one and mitochondrial mapping missed no low-intensity infections (Fig 4a-4d; S7 and S8 Files).

**Fig 4:**
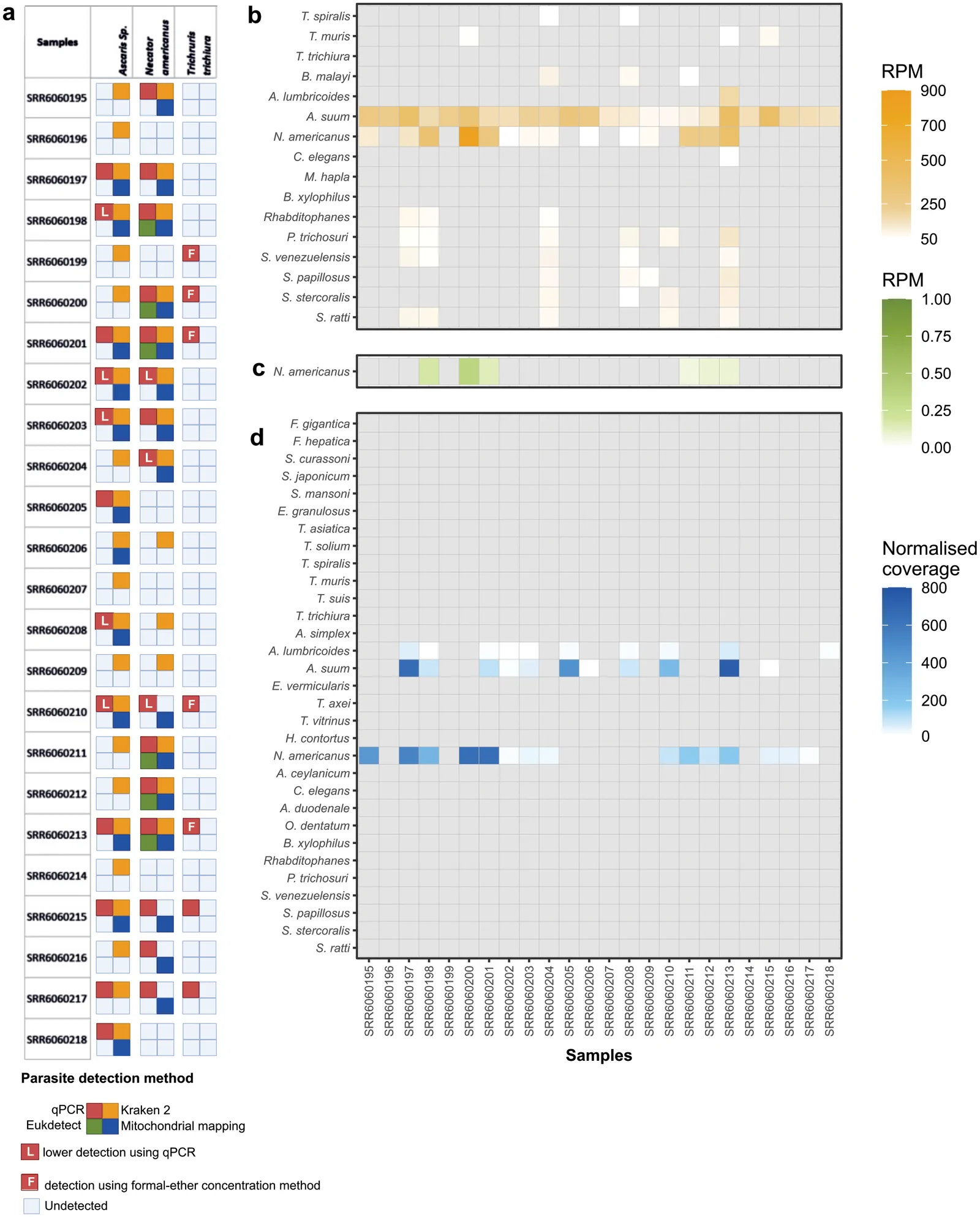
STH Detection in Human Faecal Samples. **(a)** Comparison of qPCR/formal ether concentration assays and three bioinformatics methods used on shotgun metagenomic sequences for the detection of *Ascaris spp., Necator americanus,* and *T. trichiura* in human faecal samples. Red squares indicate confirmed infection using qPCR/formal-ether concentration methods, “L” refers to samples designated as having “low” levels of detection in qPCR i.e. qPCR positive with CT > 28, “F” refers to detection using formal-ether concentration. **(b-d)** Nematode detection in SR shotgun metagenomics samples sourced from infected and uninfected human faecal samples with *Ascaris spp.*, *N. americanus* and/or *T. trichiura* using (**b**) a custom Kraken2 database containing 16 nematode genomes including the three target species with a 50 RPM threshold for detection and scaffolds <10kb removed **(c)** EukDetect marker gene detection and (**d**) mapping against 31 nematode mitochondrial genomes. RPM = Reads mapped per million reads; Normalised coverage = Reads mapped per million reads per genome size (Mb). Colour denotes level of detection, with grey squares indicating no detection. Note: *Ascaris spp.* are not included in the EukDetect database.

*Ascaris spp.* was detected in all qPCR-positive and qPCR samples negative for *Ascaris* using Kraken2 (F1 = 0.66, FPR = 100%) (Fig 4a and 4b). Mitochondrial mapping detected *Ascaris spp.* in 11 of the 12 qPCR positive samples and produced fewer false positive assignments (Fig 4a and 4d, FPR = 0.08%), providing more reliable *Ascaris spp.* detection (F1 = 0.91). Kraken2 and mitochondrial mapping successfully detected all low-level infections. Read assignments were more common for *A. suum* than *A. lumbricoides*, however, qPCR confirmation was only tested for *A. lumbricoides. Ascaris* spp. was therefore considered here at the genus level. *T. trichiura* was the only species that was not detected by either Kraken2, EukDetect, or mitochondrial genome mapping (Fig 4a-4d). Together our results support that mitochondrial mapping is the most accurate and reliable approach to identify *N. americanus* and *Ascaris spp.* infections. EukDetect had the most false negative results and Kraken2 had the most false positives (S8 File).

### SR shotgun metagenomics did not reliably detect *S. stercoralis* in all samples

The human parasite *S. stercoralis* is often associated with low or inconsistent levels of nematode material or DNA in host faeces [92,93]. To assess if SR shotgun metagenomics is suitable for detecting *S. stercoralis*, we tested seven faecal samples collected from humans in Thailand or that had recently returned from Brunei with qPCR- or microscopy-confirmed *S. stercoralis* infections.

Only three samples yielded positive results with either Kraken2 (50RPM threshold), EukDetect, and/or mitochondrial mapping (Fig 5a-5d). For the Thailand samples (CKH130, CKH21, KKU01, LWH76), the two true-positive hits corresponded to the samples with the highest infection intensity (Table 1). Kraken2 generated putative false positive assignments to multiple species (FPR = 72%, Fig 5b). Two out of seven samples assigned *S. stercoralis* correctly using all three of the analytical methods tested but four out of seven *S. stercoralis*-positive samples were not identified by SR shotgun metagenomics using any method. (Fig 5a-5d). Our results indicate that SR shotgun metagenomics is unreliable for accurate *S. stercoralis* detection based on the analytical methods we’ve used.

**Fig 5:**
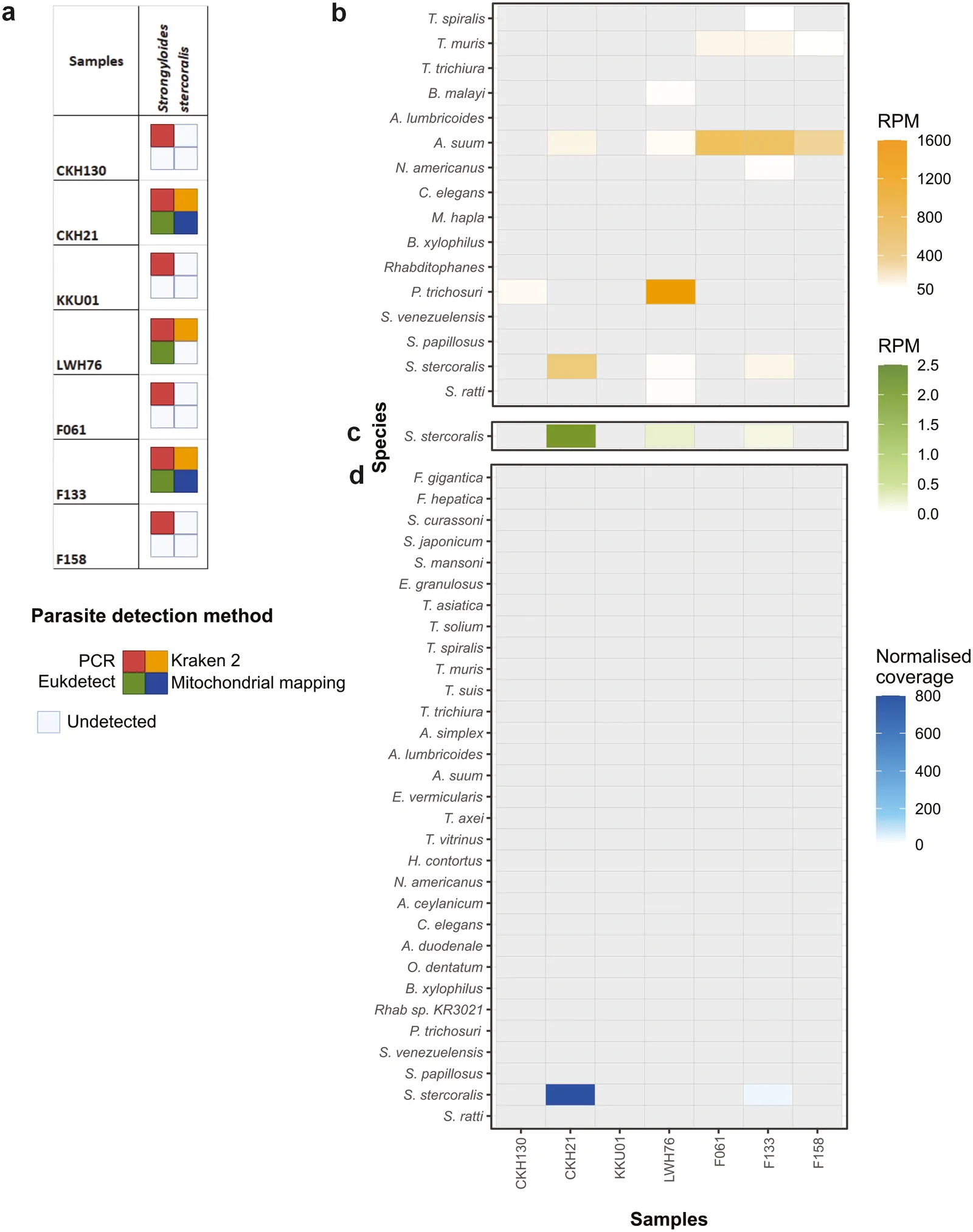
*S. stercoralis* Detection in Human Faecal Samples. (**a**) Comparison of qPCR/microscopy assays and three bioinformatics methods used on shotgun metagenomic sequences for the detection of *S. stercoralis* in human faecal samples. Red squares indicated confirmed infection. Samples CKH130, CKH21, KKU01 and LWH76 were collected from in Thailand, samples F061, F133 and F158 were collected from people travelling to the UK from Fiji. (**b-d**) Nematode detection in SR shotgun metagenomics samples sourced from individuals infected with *S. stercoralis* using (**b**) a custom Kraken2 database containing 16 nematode species, with a 50 RPM threshold for detection and scaffolds <10kb removed from consideration, (**c**) EukDetect marker gene detection and (**d**) mapping against 31 nematode mitochondrial genomes. RPM = Reads mapped per million reads; Normalised coverage = Reads mapped per million reads per genome size (Mb). Colour denotes level of detection, with grey squares indicating no detection.

### Genome assembly quality is important

Before applying Kraken2 to the human faecal samples, scaffolds <10kb were removed from the reference database because smaller scaffolds in genome assemblies often represent bacterial contamination [48]. To investigate if false positive assignments could be reduced further, we compared Kraken2 taxonomic assignment after removing scaffolds shorter than 10kb, 20kb and 40kb. The FPR of 14.2% (<10 kb) 15.3% (<20 kb) and 12.2% (40 kb) indicated that filtering of scaffolds up to 40 kb had little effect on the reducing FPR overall (S9 File).

Two samples falsely identified *P. trichosuri* (Fig 5b) and false positive assignment of *P. trichosuri* was reduced by 24% (LWH76) and 38.7% (CKH130) when scaffolds <20kb were removed, and by 68.1% (LWH76) and 81.9% (CKH130), when scaffolds <40kb were removed. However, false positive assignment to *P. trichosuri* was still higher than alignments to *S. stercoralis*, indicating that removal of longer scaffolds did improve taxonomic assignment of *P. trichosuri*, but was insufficient for reliable STH detection (S9 File). The sample with the highest false assignment to *P. trichosuri* (LWH76) aligned 180,246 reads to 530 of the 1,810 scaffolds in the *P. trichosuri* genome. Of these, 524 scaffolds were <40 kb in length, and the longest scaffold was 1.04 Mb (S2 Fig, S9 File). Overall, 93.4% of *P. trichosuri*-aligned reads were of bacterial origin with 91.22% mean sequence identity to *Brevundimonas* spp., which are ubiquitous in the environment (S3 Fig, S9 File). These results indicate that false positive alignments occur due to contamination from bacterial sequences incorporated into eukaryotic genome assemblies.

## Discussion

Shotgun metagenomics has potential for detecting STH infections and understanding microbiota-STH interactions [49,51,52,55,67–69]. Method validation is essential for accurate taxonomic assignment, maximising sensitivity and specificity for the application of shotgun metagenomics as a diagnostic tool. We have compared the analytical approach and sequencing technologies for shotgun metagenomics for two infection intensities in a laboratory model of STH infection using *S. ratti*, and tested human faecal samples with confirmed (using qPCR or formol-ether concentration) infections for at least one of four STHs.

Mitochondrial mapping of SR metagenomics samples was the most accurate method of STH detection in rats infected with *S. ratti*, Other methods based on k-mer identification (Kraken2), marker genes (EukDetect) and protein sequences (DIAMOND+MEGAN) performed less well at a lower infection intensity. These results were mirrored in STH-infected human samples, where mitochondrial genome mapping was consistently the most effective method at minimising false positive and false negative detection in faecal samples infected with *Ascaris spp* and *N. americanus*. This supports previous work where mitochondrial mapping outperformed other methods of taxonomic assignment and likely reflects the high copy numbers of the mitochondrial genome present, thus increasing the chance of detection [48,91].

The enhanced performance of mitochondrial mapping may also reflect that mitochondrial genomes are often more complete and better curated than nuclear assemblies for many parasitic species, and they are present in higher copy numbers than nuclear genomes. The mitochondrial mapping method is limited to detection of species with available mitochondrial genome sequences. While mitochondrial data are largely available for medically important parasites such as STHs due to sampling bias of medically and economically important parasites, this could be problematic for broader applications such as for helminth parasites that are less prevalent or specifically infect wildlife. In 2024, there were complete mitochondrial genomes for 255 nematodes, 126 cestodes, 101 trematodes, and 24 acanthocephalan species available on NCBI, but up to 350,000 helminth species are estimated to exist [94]. Where mitochondrial genomes are not available, caution should be taken when interpreting taxonomic assignments.

Nuclear genomes of helminths can be fragmented, contaminated with microbial sequences or incomplete, which can reduce the sensitivity and specificity of k-mer-, marker-, or protein-based detection methods. This discrepancy underscores the need for more complete and well-annotated nuclear genome assemblies if shotgun metagenomics is to be used as a reliable and generalisable detection strategy across diverse parasite taxa. False positive identification due to poor quality genome assemblies is an issue and poses a barrier to using shotgun metagenomics for detecting eukaryotes in primarily bacterial environments. Where eukaryote reference genomes contain bacterial contamination from common environmental sources, such as the *Brevundimonas* contamination in the *P. trichosuri* genome, signals for the true positive species present may be negligible. Bacterial contamination in eukaryotic genomes is increasingly documented [48,95,96] and could also be responsible for *E. vermicularis* false positives in our laboratory samples. While removing smaller scaffolds from reference databases does improve false positive detection rates, it does not resolve the issue. Continued efforts to refine and curate reference genomes will benefit shotgun metagenomics studies concerned with accurate identification of eukaryotes. In general, STH genome assemblies have received more attention than other helminths and are therefore of a higher standard. However, awareness of the quality of data in reference databases is important for meaningful interpretation of results.

A remaining problem is that only a small proportion of the DNA, and subsequently the sequenced reads to be analysed in samples, are of parasite origin. In samples where DNA levels are expected to be low, detection was unreliable. Specifically, (i) in the *S. ratti* laboratory system, low infection intensity was difficult to detect using 3 out of 4 methods tested, (ii) *T. trichiura* could not be detected in human faecal samples - *T. trichiura* eggs are resilient compared to other STHs [97], which can lead to difficulties in releasing DNA, e.g., a previous attempt to detect *T. trichiura* using metagenomics obtained few reads [98]; and (iii) *S. stercoralis* was more readily detected in samples with higher larval recovery. Together these results support that the level of STH material, and thus DNA present in samples is important for shotgun-based STH detection. In addition to infection intensity, numerous species-specific factors influence the level of STH and associated DNA in a sample including genome size, DNA accessibility (e.g., some eggs are more resistant to lysis and DNA extraction), developmental stage of eggs or larvae, temporal variation in STH shedding patterns, and target gene copy number. Recent work by Papaiakovou *et al* (2025) also highlights the importance of genetic variation at the population level [54]. These factors are further confounded by heterogeneity of material in faecal samples, sampling methods and differences in DNA extraction protocols. Future efforts focusing on enhancing recovery of parasite material before DNA extraction or during sequencing could resolve this. Development of laboratory based methods, not explored here, to concentrate parasite material and DNA recovery could further aide detection methods. Recent studies have investigated approaches to increase parasite-derived DNA signal in complex samples, notably through hybridization capture [99] and refinement of metabarcoding and library-preparation workflows [100]. These enrichment and targeted sequencing techniques highlight promising avenues for improving parasite detection sensitivity in shotgun metagenomics. These methods will still require comprehensive validation such as benchmarking against confirmed infections and assessment of specificity and sensitivity.

LR metagenomic sequencing approaches including shotgun metagenomics and LR amplicon sequencing and LR hybrid capture methodologies, have potential to distinguish deeper taxonomic levels such as at a strain level, or could provide additional information about the diversity of parasite populations [101]. This could aid detection of less well characterised parasites or where genetic diversity could impair diagnostics. LRs can improve taxonomic recall, but do not appear to eliminate false positive assignments [102]. However, the ONT metagenomics used here did not reliably detect *S. ratti* true-positives. Hybrid approaches, which combine short and long read data successfully detected true-positives and no false positives, but this was mostly attributed to the SRs present, thus offering no benefit of LR sequencing. LR data analysis for eukaryotic metagenomics requires further development to unlock any potential benefits. However, currently the increased computational complexity and analysis time, high cost, higher input levels and generation of low read numbers further impacts feasibility for large-scale or resource-limited studies with LR metagenomics, compared to short read metagenomic. The development of more accurate and computationally efficient tools tailored to LR eukaryotic metagenomic data should be a priority to fully realise the potential of LR and hybrid sequencing for STH detection.

## Supporting information

S1 Methods Supplementary methods. (DOCX)

S1 Fig Experimental design, sequencing approaches, and bioinformatic approaches used for parasite detection in human and rat samples. (DOCX)

S2 Fig Coverage of LWH76 alignments to the *P. trichosuri* Genome show that the Majority of Hits are to Small Scaffolds. (DOCX)

S3 Fig Alignment of *P. trichosuri* Contigs to the *B. naejangsanensis* Genome Indicates Bacterial Contamination. (DOCX)

S1 Table Standard Dose/Low Sequencing Coverage. Short read metagenomics of rat faecal samples.
(DOCX)

S2 Table Low Dose/Low Sequencing Coverage. Short read metagenomics of rat faecal samples.
(DOCX)

S3 Table Standard Dose/High Sequencing Coverage. Short read metagenomics of rat faecal samples.
(DOCX)

S4 Table Short read metagenomic shotgun reads sequenced from human samples with positive PCR for *Strongyloides stercoralis* infection. (DOCX)

S5 Table Long read metagenomics of rat faecal samples - raw sequencing data and host removed/filtered sequence data. (DOCX)

S6 Table Parasite genomes included in custom Kraken2 database. (DOCX)

S7 Table Hybrid Assembly Metrics. (DOCX)

S8 Table Short read metagenomic shotgun reads. (DOCX)

S9 Table Larval counts for S. ratti infectiosn at 6 and 11 days post-infection at standard and low infection intensities. (DOCX)

S1 File Coverage values and reads aligned per million reads from Kraken2, DIAMOND+MEGAN. EukDetect and mitochondrial mapping using SR metagenomics.
(XLSX)

S2 File Taxonomic assignments for long read data reported as Bases per million and normalised coverage values. (XLSX)

S3 File Taxonomic assignment of binned sequences from MetaBAT2. (XLSX)

S4 File BLAST results of the unbinned sequences. (XLSX)

S5 File Measures of sensitivity, specificity, precision, false positive detection rate (FPR), and F1 scores for SR analysis. (XLSX)

S6 File Measures of sensitivity, specificity, precision, false positive detection rate (FPR), and F1 scores LR and long-short read hybrid analysis. (XLSX)

S7 File Coverage values and reads aligned per million reads from Kraken2, DIAMOND+MEGAN. EukDetect and mitochondrial mapping using,etagenomic shotgun human samples.
(XLSX)

S8 File Measures of sensitivity, specificity, precision, false positive detection rate (FPR), and F1 scores from human samples. (XLSX)

S9 File Kraken2 taxonomic assignment after removing scaffolds shorter than 10kb, 20kb and 40kb. The FPR of 14.2% (<10 kb) 15.3% (<20 kb) and 12.2% (40 kb).
(XLSX)
