# Supplementary material for: Hunting for Helminths: short- and long-read shotgun metagenomics for helminth detection in faecal samples
Source: PLoS Negl Trop Dis. 2026 Sep 21;20(9):e0014130. doi: 10.1371/journal.pntd.0014130 (PMC13645222; doi:10.1371/journal.pntd.0014130)
Supplement: Supplementary file 1 [file pntd.0014130.s001.docx]

**S1 Methods**

**DNA extraction**

200 mg aliquots of faecal samples were thawed on ice and added to a lysing tube (Lysing Matrix E, 2mL tubes, MP Bio) containing 1.4mm ceramic spheres, 0.1 mm silica spheres and one 4mm glass bead. Samples were homogenised vigorously using a bead beater at 5000 RPM for 3 x 20 seconds with 60 second intervals. DNA was extracted using the QIAamp PowerFecal Pro DNA kit (Qiagen) according to the manufacturer’s instructions.

## **Sample collection for *Strongyloides stercoralis* infection**

### Faecal sample collection and analysis

i. **Thailand samples**

Individuals provided fresh stool which were transported to the Tropical Disease Research Centre, Faculty of Medicine, Khon Kaen University in Thailand within two hours of collection. Samples CKH130, CKH21 and LWH76 were collected from a single rural site, and sample KKU01 was collected from a hospital patient who tested positive for *Strongyloides* (**Table 1**). To detect *Strongyloides* infection and count infective third-stage larvae (iL3s), fresh faeces were cultured individually as described by ^86^. After 72 hours of culture, the faecal sample were removed and the liquid contents were examined under a stereo microscope (Zeiss) (**Table 1**). This study was approved by the Khon Kaen University Ethics Committee for Human Research (reference HE641605) and the Central University Research Ethics Committee at the University of Liverpool (reference 10936). All patients in Thailand who tested positive for *Strongyloides* infection were provided with Albendazole as an anthelmintic treatment. DNA extraction was carried out as described above.

ii. **Fijian migrant samples collected in the UK**

After giving written informed consent, stool was obtained from three *Strongyloides* positive individuals who had migrated to the UK from Fiji. Samples were transported unrefrigerated and processed within 18-24 hours of passage at the Clinical Diagnostic Parasitology Laboratory at the Liverpool School of Tropical Medicine. All three samples were positive for *Strongyloides* infection using multiplex qPCR. DNA was extracted from samples as described by Cunningham, et al. ^87^ ^88^. In brief, 0.1 mg of faeces was added to a 2 ml screwcap tube containing 0.9 mg of 1.4 mm ceramic beads (Qiagen, Manchester, UK). To this the PVPP DNA (Merck, Gillingham, UK) extraction buffer was added, and the sample was frozen overnight at -20°C. Samples were allowed to defrost and underwent bead-beating at 3000 rpm for 30 seconds using the MagnaLyser (Roche, Burgess Hill, UK) system. Following bead-beating, samples were processed using the QIAmp DNA Mini Kit with the following modifications to the protocol: 1) 1µL of phocine herpes virus-1 (PhHv1), per-sample, was added to the AL buffer, to act as an internal positive control and 2) the DNA was eluted into 100uL of AE buffer. qPCR for *Strongyloides* was performed using published primers ^89^ on the Rotor-Gene platform (Qiagen, Manchester, UK). For a single 20µL reaction, 2µL of DNA template and 10µL of Bio Rad iQ™ Supermix (Bio-Rad, Watford, UK) was used. The thermocycler conditions were as follows: hold at 95°C for 15 minutes; then 50 cycles of 94°C for 15 seconds, followed by 60°C for 60 seconds. A clinical Cycle threshold (Ct) of <38 were considered positive. All three participants provided serum samples which tested positive using a commercial *Strongyloides stercoralis* IgG ELISA (DRG Instruments, Marburg, Germany) and had an eosinophilia, defined as an eosinophil count > 0.5 x 10^9^/L.

### **Bacterial contamination of genome assemblies**

The custom Kraken2 database was modified to remove nematode reference genome sequences i) <10kb, (ii) <20kb, and iii) <40kb in length. We aligned reads to the *P. trichosuri* genome using Bowtie2. BAM QC analysis from Qualimap v2.3 was run on the BAM file with default parameters ^90^. Aligned reads were extracted using Bedtools v.2.29.2 ‘bamtofastq’ and classified using the Kraken2 Minikraken DB v.1 (Refseq: bacteria, archaea, viral; <https://benlangmead.github.io/aws-indexes/>). Bacterial contaminants were identified by assessing the percentage of reads assigned to each bacterial species based on the MiniKraken alignment results. Bacterial species representing more than 3% of the total assigned reads was considered a potential contaminant. A custom bacterial BLAST database was generated using the ‘makeblastdb’ (Blast+ v.2.9.0) command with default parameters, comprising the genomes of the identified bacterial contaminants. Nematode genomes were queried against the custom bacterial BLAST database and results visualised using Kablammo ^91^. A sequence alignment with <10bp difference between query and subject length was considered a full-length alignment.

**Comparisons of computational effort between Kraken2, DIAMOND+MEGAN and EukDetect**

As the computational resources available to each researcher are different, we aimed to provide an estimation of computing time per method using the following setup:

- Operating System: Linux
- Kernal Version: 5.15.146.1-microsoft-standard-WSL2
- Architecture: x86_64 (64-bit)
- CPU Model: Intel(R) Core(TM) i9-10900X CPU @ 3.70GHz
- CPU Cores: 20
- Total memory (RAM): 62 GB

**Kraken2**

Building the Kraken2 database was the most time-consuming part of this analysis, as construction took ~5 hours. Classification was faster, with an average time of 15.38 minutes per sample for low dose samples and 11.30 minutes for standard dose samples.

**DIAMOND+MEGAN**

Computational effort was prohibitively high for the DIAMOND+MEGAN pipeline compared to other methods. Downloading the nr database took 4.88 hours, and average time for DIAMOND to complete was 48 hours per sample for the standard dose dataset and 72.17 hours per sample for the low dose dataset. In addition, diamond output files had to be meganized, which took on average 1.89 hours per sample for standard dose, and 1.67 hours per sample for low dose samples. Re-running the DIAMOND+MEGAN pipeline on extracted eukaryotic reads was far less computationally intensive, with an average time of 0.87 hours per sample for the standard dose dataset and 0.64 hours per sample for the low dose dataset.

**EukDetect**

Computationally, EukDetect was one of the least time-consuming of the four methods we compared, as the database required was much smaller (3.12 GB). Additionally, running the pipeline was faster, with an average of 0.9 and 2.3 minutes per sample for the standard dose, and low dose samples, respectively.

**Mitochondrial Mapping**

Mitochondrial mapping was also less time-consuming than Kraken2 or DIAMOND+MEGAN, as it involved manually downloading mitochondrial sequences from NCBI (~60 minutes) and following the methods described in (Papaiakovou et al. 2023). The database required for this study was 440KB, and files were processed with an average of 23 and 25 minutes for standard dose, and low dose samples, respectively. Script for mitochondrial mapping can be found here - <https://github.com/MarinaSci/IJP_genome_skimming>
