# Supplementary material for: Hunting for Helminths: short- and long-read shotgun metagenomics for helminth detection in faecal samples
Source: PLoS Negl Trop Dis. 2026 Sep 21;20(9):e0014130. doi: 10.1371/journal.pntd.0014130 (PMC13645222; doi:10.1371/journal.pntd.0014130)
Supplement: Supplementary file 2 [file pntd.0014130.s002.docx]

# **S1 Fig: Experimental design, sequencing approaches, and bioinformatic approaches used for parasite detection in human and rat samples.**

S1 Fig : Workflow summarising sample collection, sequencing methodologies, number of replicates and downstream bioinformatic approaches used for A) rat derived and B) human derived faecal samples.
