# Supplementary material for: Hunting for Helminths: short- and long-read shotgun metagenomics for helminth detection in faecal samples
Source: PLoS Negl Trop Dis. 2026 Sep 21;20(9):e0014130. doi: 10.1371/journal.pntd.0014130 (PMC13645222; doi:10.1371/journal.pntd.0014130)
Supplement: Supplementary file 3 [file pntd.0014130.s003.docx]

**S2 Fig** : **Coverage of LWH76 alignments to the *P. trichosuri* Genome show that the Majority of Hits are to Small Scaffolds.** Coverage depth and GC content distribution of short-read shotgun metagenomics reads aligned to the P. trichosuri reference genome for (**a**) LWH76. In the top panel the y-axis represents the coverage depth, and the x-axis represents the position along the *P. trichosuri* reference genome in base pairs (bp), with the size of the scaffolds decreasing from left to right. Red shading towards the right side of the plot corresponds to smaller contigs/scaffolds of the genome, which exhibit significantly higher coverage compared to other regions. In the bottom panel, the y-axis shows GC content across the *P. trichosuri* genome (black line) and its average value (red dotted line) and the x-axis represents the position along the *P. trichosuri* reference genome in base pairs (bp).
