# Supplementary material for: Hunting for Helminths: short- and long-read shotgun metagenomics for helminth detection in faecal samples
Source: PLoS Negl Trop Dis. 2026 Sep 21;20(9):e0014130. doi: 10.1371/journal.pntd.0014130 (PMC13645222; doi:10.1371/journal.pntd.0014130)
Supplement: Supplementary file 4 [file pntd.0014130.s004.docx]

**S3 Fig** : **Alignment of *P. trichosuri* Contigs to the *B. naejangsanensis* Genome Indicates Bacterial Contamination.** (**a-c**) Top three highest bit score alignments between *P. trichosuri* contigs and the *B. naejangsanensis* genome. In each panel, the top line is the query sequence (*P. trichosuri* contigs), and the bottom line shows the corresponding section of the *B. naejangsanensis* genome (Bacteria). Blue boxes show highly similar regions between the query and the bacterial genome.
