# Supplementary material for: Hunting for Helminths: short- and long-read shotgun metagenomics for helminth detection in faecal samples
Source: PLoS Negl Trop Dis. 2026 Sep 21;20(9):e0014130. doi: 10.1371/journal.pntd.0014130 (PMC13645222; doi:10.1371/journal.pntd.0014130)
Supplement: Supplementary file 5 [file pntd.0014130.s005.docx]

**S1 Table : Standard Dose/Low Sequencing Coverage**

| Sample | Total Reads | Host Removed Sequences |
| --- | --- | --- |
| 11dpi.1 | 10,320,023 | 10,256,947 (99.39%) |
| 11dpi.2 | 11,986,703 | 11,958,766 (99.77%) |
| 11dpi.3 | 9,252,283 | 9,185,516 (99.28%) |
| 6dpi.1 | 12,863,414 | 12,736,653 (99.01%) |
| 6dpi.2 | 9,253,414 | 9,220,989 (99.65%) |
| 6dpi.3 | 8,839,452 | 8,826,255 (99.85%) |
| 11dpi_Control.1 | 9,796,174 | 9,349,969 (95.45%) |
| 11dpi_Control.2 | 12,589,361 | 12,493,540 (99.24%) |
| 11dpi_Control.3 | 9,266,163 | 9,215,483 (99.45%) |
| 6dpi_Control.1 | 12,404,315 | 12,322,408 (99.34%) |
| 6dpi_Control.2 | 9,543,081 | 9,445,203 (98.97%) |
| 6dpi_Control.3 | 9,388,397 | 9,300,187 (99.06%) |
