# Supplementary material for: Hunting for Helminths: short- and long-read shotgun metagenomics for helminth detection in faecal samples
Source: PLoS Negl Trop Dis. 2026 Sep 21;20(9):e0014130. doi: 10.1371/journal.pntd.0014130 (PMC13645222; doi:10.1371/journal.pntd.0014130)
Supplement: Supplementary file 6 [file pntd.0014130.s006.docx]

**S2 Table : Low Dose/Low Sequencing Coverage**

| Sample | Total Reads | Host Removed Sequences |
| --- | --- | --- |
| 11dpi.1 | 10,716,179 | 10,709,490 (99.94%) |
| 11dpi.2 | 15,527,154 | 15,477,194 (99.68%) |
| 11dpi.3 | 11,827,996 | 11,731,897 (99.19%) |
| 6dpi.1 | 12,777,791 | 12,711,133 (99.48%) |
| 6dpi.2 | 13,279,348 | 13,261,790 (99.87%) |
| 6dpi.3 | 13,296,795 | 13,265,357 (99.76%) |
| Control.1 | 13,358,193 | 13,318,874 (99.71%) |
| Control.2 | 12,811,843 | 12,803,482 (99.93%) |
| Control.3 | 12,669,748 | 12,645,556 (99.81%) |
