# Supplementary material for: Hunting for Helminths: short- and long-read shotgun metagenomics for helminth detection in faecal samples
Source: PLoS Negl Trop Dis. 2026 Sep 21;20(9):e0014130. doi: 10.1371/journal.pntd.0014130 (PMC13645222; doi:10.1371/journal.pntd.0014130)
Supplement: Supplementary file 7 [file pntd.0014130.s007.docx]

**S3 Table : Standard Dose/High Sequencing Coverage**

| Sample | Total Reads | Host Removed Sequences |
| --- | --- | --- |
| 11dpi.1 | 59,512,969 | 59,134,383 (99.36%) |
| 11dpi.2 | 67,666,800 | 67,507,252 (99.76%) |
| 11dpi.3 | 54,396,828 | 53,983,436 (99.24%) |
| 6dpi.1 | 72,568,678 | 71,830,833 (98.98%) |
| 6dpi.2 | 54,955,887 | 54,758,666 (99.64%) |
| 6dpi.3 | 53,415,787 | 53,339,037 (99.86%) |
| 11dpi_Control.1 | 59,206,201 | 56,379,206 (95.23%) |
| 11dpi_Control.2 | 73,296,252 | 72,717,528 (99.21%) |
| 11dpi_Control.3 | 53,271,738 | 52,974,658 (99.44%) |
| 6dpi_Control.1 | 70,198,171 | 69,720,093 (99.32%) |
| 6dpi_Control.2 | 56,979,984 | 56,383,501 (98.95%) |
| 6dpi_Control.3 | 55,308,203 | 54,771,241 (99.03%) |
