# Supplementary material for: Hunting for Helminths: short- and long-read shotgun metagenomics for helminth detection in faecal samples
Source: PLoS Negl Trop Dis. 2026 Sep 21;20(9):e0014130. doi: 10.1371/journal.pntd.0014130 (PMC13645222; doi:10.1371/journal.pntd.0014130)
Supplement: Supplementary file 8 [file pntd.0014130.s008.docx]

**S4 Table: Short-read metagenomic shotgun reads sequenced from human samples with positive PCR for *Strongyloides stercoralis* infection – raw sequencing data and host read removal.**

| Sample | Origin | Total Reads | Host Removed Sequences |
| --- | --- | --- | --- |
| CKH130 | Thailand | 22,898,316 | 22,898,133 (99.99%) |
| CKH21 | Thailand | 45,332,686 | 45,330,515 (99.99%) |
| KKU01 | Thailand | 34,555,157 | 34,551,937 (99.99%) |
| LWH76 | Thailand | 21,611,975 | 21,610,848 (99.99%) |
| F25199492 | UK | 19,780,957 | 19,744,848 (99.82%) |
| F30368900 | UK | 21,811,043 | 21,804,186 (99.97%) |
| F30371358 | UK | 20,509,037 | 20,500,488 (99.96%) |
