# Supplementary material for: Hunting for Helminths: short- and long-read shotgun metagenomics for helminth detection in faecal samples
Source: PLoS Negl Trop Dis. 2026 Sep 21;20(9):e0014130. doi: 10.1371/journal.pntd.0014130 (PMC13645222; doi:10.1371/journal.pntd.0014130)
Supplement: Supplementary file 9 [file pntd.0014130.s009.docx]

**S5 Table : Long-read metagenomics of rat faecal samples - raw sequencing data and host removed/filtered sequence data**

| Sample | Total Sequences | Sequence Length Distribution (bp) | Host Removed + Filtered (Gb) |
| --- | --- | --- | --- |
| Standard_dose | 5,760,121 | 87 - 336,354 | 13.81 |
| Low_dose | 8,611,209 | 92 - 79,484 | 16.58 |
| Control | 11,277,610 | 90 - 72,624 | 18.40 |
