# Supplementary material for: Hunting for Helminths: short- and long-read shotgun metagenomics for helminth detection in faecal samples
Source: PLoS Negl Trop Dis. 2026 Sep 21;20(9):e0014130. doi: 10.1371/journal.pntd.0014130 (PMC13645222; doi:10.1371/journal.pntd.0014130)
Supplement: Supplementary file 10 [file pntd.0014130.s010.docx]

**S6 Table : Parasite genomes included in custom Kraken2 database.**

| Species | Genome Size | Scaffold Length | Longest Scaffold | N50 Length | N90 Length |
| --- | --- | --- | --- | --- | --- |
| *Strongyloides ratti* | 43,166,851 | 43 Mb | 16.8 Mb | 11.7 Mb | 748.2 kb |
| *Strongyloides stercoralis* | 42,674,647 | 43 Mb | 5.0 Mb | 431.1 kb | 96.2 kb |
| *Parastrongyloides trichosuri* | 42,486,966 | 42 Mb | 6.2 Mb | 836.9 kb | 13.4 kb |
| *Strongyloides papillosus* | 60,447,969 | 60 Mb | 1.7 Mb | 86.4 kb | 4.0 kb |
| *Strongyloides venezuelensis* | 52,178,999 | 52 Mb | 5.9 Mb | 715.4 kb | 109.8 kb |
| *Rhabditophanes sp. KR3021* | 47,267,908 | 47 Mb | 7.3 Mb | 537.2 kb | 79.2 kb |
| *Meloidogyne hapla* | 53,017,507 | 53 Mb | 360.4 kb | 37.6 kb | 6.5 kb |
| *Bursaphelenchus xylophilus* | 74,561,461 | 75 Mb | 3.6 Mb | 949.8 kb | 4.3 kb |
| *Ascaris suum* | 298,028,455 | 298 Mb | 13.4 Mb | 4.6 Mb | 918.1 kb |
| *Brugia malayi* | 88,235,797 | 88 Mb | 24.9 Mb | 14.2 Mb | 13.5 Mb |
| *Trichuris muris* | 111,837,642 | 112 Mb | 36.4 Mb | 28.9 Mb | 37.7 kb |
| *Trichinella spiralis* | 63,525,422 | 64 Mb | 12.0 Mb | 6.4 Mb | 2.0 kb |
| *Necator americanus* | 244,075,060 | 244 Mb | 1.9 Mb | 211.9 kb | 29.2 kb |
| *Caenorhabditis elegans* | 100,286,401 | 100 Mb | 20.9 Mb | 17.5 Mb | 13.8 Mb |
| *Ascaris lumbricoides** | 316,975,410 | 316 Mb | 1.0 Mb | 56 kb | 5.01 kb |
| *Trichuris trichiura** | 8,05,73,711 | 80 Mb | 29.1 Mb | 11.2 Mb | 643.7 kb |

*These genomes were only added to custom Kraken2 database prior to investigation of human field samples infected with *Ascaris lumbricoides* or *Trichuris trichiura*
