# Supplementary material for: Hunting for Helminths: short- and long-read shotgun metagenomics for helminth detection in faecal samples
Source: PLoS Negl Trop Dis. 2026 Sep 21;20(9):e0014130. doi: 10.1371/journal.pntd.0014130 (PMC13645222; doi:10.1371/journal.pntd.0014130)
Supplement: Supplementary file 11 [file pntd.0014130.s011.docx]

**S7 Table : Hybrid Assembly Metrics**

| Metric | Control | Low_Dose | Standard_Dose |
| --- | --- | --- | --- |
| Assembly Total Length (bp) | 435,436,003 | 521,310,794 | 1,137,183,161 |
| # Contigs | 445,183 | 552,004 | 1,399,113 |
| Largest Contig (bp) | 1,283,117 | 3,368,289 | 2,160,277 |
| Assembly Size (Mb) | 435.4 | 521.3 | 1,137.1 |
| GC (%) | 48.85 | 48.04 | 47.75 |
| N50 | 12,714 | 10,105 | 8,822 |
| N90 | 1,430 | 1,496 | 817 |
| # Bins | 143 | 165 | 291 |
| Complete Genomes (<10% Contamination, >90% Completeness) | 15 | 15 | 47 |
