# Supplementary material for: Hunting for Helminths: short- and long-read shotgun metagenomics for helminth detection in faecal samples
Source: PLoS Negl Trop Dis. 2026 Sep 21;20(9):e0014130. doi: 10.1371/journal.pntd.0014130 (PMC13645222; doi:10.1371/journal.pntd.0014130)
Supplement: Supplementary file 12 [file pntd.0014130.s012.docx]

**S8 Table : Short-read metagenomic shotgun reads sourced from (Rosa et al. 2018) and qPCR results.**

| **Sample** | **Ascaris lumbricoides** | **Necator americanus** | **Trichuris trichiura** |
| --- | --- | --- | --- |
| SRR6060195 | - | Y | - |
| SRR6060196 | - | - | - |
| SRR6060197 | Y | Y | - |
| SRR6060198 | LOW | Y | - |
| SRR6060199 | - | - | Y* |
| SRR6060200 | - | Y | Y* |
| SRR6060201 | Y | Y | Y* |
| SRR6060202 | LOW | LOW | - |
| SRR6060203 | LOW | Y | - |
| SRR6060204 | - | LOW | - |
| SRR6060205 | Y | - | - |
| SRR6060206 | - | - | - |
| SRR6060207 | - | - | - |
| SRR6060208 | LOW | - | - |
| SRR6060209 | - | - | - |
| SRR6060210 | LOW | LOW | Y* |
| SRR6060211 | - | Y | - |
| SRR6060212 | - | Y | - |
| SRR6060213 | Y | Y | Y* |
| SRR6060214 | - | - | - |
| SRR6060215 | Y | Y | Y |
| SRR6060216 | - | Y | - |
| SRR6060217 | Y | Y | Y |
| SRR6060218 | Y | - | - |

**Y = confirmed infection using qPCR, Y* = confirmed infection using formal-ether concentration method, LOW = low level detection using qPCR**
