# Supplementary material for: Hunting for Helminths: short- and long-read shotgun metagenomics for helminth detection in faecal samples
Source: PLoS Negl Trop Dis. 2026 Sep 21;20(9):e0014130. doi: 10.1371/journal.pntd.0014130 (PMC13645222; doi:10.1371/journal.pntd.0014130)
Supplement: Supplementary file 13 [file pntd.0014130.s013.docx]

**S9 Table : Larval counts for *S. ratti* infection for standard and low infection intensities.**

| No. iL3's infected | Days post infection | Replicate | No. of L1s recovered by Baermann funnel | L1s per gram of faeces |
| --- | --- | --- | --- | --- |
| 50 | 6 | 1 | 300 | 37.5 |
| 50 | 6 | 2 | 300 | 25 |
| 50 | 11 | 1 | 67 | 8.4 |
| 50 | 11 | 2 | 200 | 15.4 |
| 500 | 11 | 1 | 2733 | 273.3 |
| 500 | 11 | 2 | 2000 | 142.9 |
| 500 | 6 | 1 | 1033 | 114.8 |
| 500 | 6 | 2 | 833 | 92.6 |
